# Detection and Quantification of *Mycobacterium tuberculosis* in the Sputum of Culture-Negative HIV-infected Pulmonary Tuberculosis Suspects: A Proof-of-Concept Study

**DOI:** 10.1371/journal.pone.0158371

**Published:** 2016-07-08

**Authors:** Guillermo Madico, Moses Mpeirwe, Laura White, Solange Vinhas, Beverley Orr, Patrick Orikiriza, Nancy S. Miller, Mary Gaeddert, Juliet Mwanga-Amumpaire, Moises Palaci, Barry Kreiswirth, Joe Straight, Reynaldo Dietze, Yap Boum, Edward C. Jones-López

**Affiliations:** 1 Section of Infectious Diseases, Department of Medicine, Boston Medical Center and Boston University School of Medicine, Boston, Massachusetts, United States of America; 2 Epicentre, Médecins sans Frontières, Mbarara, Uganda; 3 Mbarara University of Science and Technology, Mbarara, Uganda; 4 Department of Biostatistics, Boston University School of Public Health, Boston, Massachusetts, United States of America; 5 Núcleo de Doenças Infecciosas, Universidade Federal do Espírito Santo, Vitória, Brazil; 6 Clinical Microbiology Laboratory, Boston Medical Center, Boston, Massachusetts, United States of America; 7 Department of Pathology and Laboratory Medicine, Boston University School of Medicine, Boston, Massachusetts, United States of America; 8 Public Health Research Institute (PHRI) – Rutgers University, Newark, New Jersey, United States of America; 9 Thisis Diagnostics Inc., Boston, Massachusetts, United States of America; McGill University, CANADA

## Abstract

**Rationale:**

Rapid diagnosis of pulmonary tuberculosis (TB) is critical for timely initiation of treatment and interruption of transmission. Yet, despite recent advances, many patients remain undiagnosed. Culture, usually considered the most sensitive diagnostic method, is sub-optimal for paucibacillary disease.

**Methods:**

We evaluated the Totally Optimized PCR (TOP) TB assay, a new molecular test that we hypothesize is more sensitive than culture. After pre-clinical studies, we estimated TOP’s per-patient sensitivity and specificity in a convenience sample of 261 HIV-infected pulmonary TB suspects enrolled into a TB diagnostic study in Mbarara, Uganda against MGIT culture, Xpert MTB/RIF and a composite reference standard. We validated results with a confirmatory PCR used for sequencing *M*. *tuberculosis*.

**Measurements and Results:**

Using culture as reference, TOP had 100% sensitivity but 35% specificity. Against a composite reference standard, the sensitivity of culture (27%) and Xpert MTB/RIF (27%) was lower than TOP (99%), with similar specificity (100%, 98% and 87%, respectively). In unadjusted analyses, culture-negative/TOP-positive patients were more likely to be older (P<0·001), female (P<0·001), have salivary sputum (P = 0·05), sputum smear-negative (P<0.001) and less advanced disease on chest radiograph (P = 0.05). *M*. *tuberculosis* genotypes identified in sputum by DNA sequencing exhibit differential growth in culture.

**Conclusions:**

These findings suggest that the TOP TB assay is accurately detecting *M*. *tuberculosis* DNA in the sputum of culture-negative tuberculosis suspects. Our results require prospective validation with clinical outcomes. If the operating characteristics of the TOP assay are confirmed in future studies, it will be justified as a “TB rule out” test.

## Introduction

Despite recent advances, tuberculosis (TB) remains a major global health problem with 9 million new cases and 1.4 million deaths in 2013.[1] Critically, the global incidence is decreasing by less than 2% per year, far from the 20% decline required to reach the World Health Organization (WHO) stated goal of eliminating TB by 2050.[2, 3] Patients with pulmonary TB represent ~75% of the global disease burden and contribute exclusively to transmission. Rapid, accurate and early detection of *Mycobacterium tuberculosis* (MTB) in the sputum of TB suspects, and active case finding are key components of the WHO strategy.[4, 5]

For decades, the rapid diagnosis of pulmonary TB has relied on sputum acid-fast bacilli (AFB) smear microscopy but its yield is low when compared to mycobacterial culture, which is considered the most sensitive method for diagnosis.[6] Recently developed molecular tests such as Xpert^®^ MTB/RIF and GenoType^®^ MTBDRplus provide a rapid alternative to culture in patients with high bacterial loads (i.e. sputum AFB smear-positive). However, their overall sensitivity (~90% against culture) in programmatic conditions has been lower than initially anticipated,[7] and particularly poor (~50%) in smear-negative/culture-positive individuals.[8–10] Other TB diagnostics under development suffer the common limitation of being less sensitive than cultures.[5, 11, 12]

For definitive diagnosis, reliance on cultures as the reference method is problematic because the process of decontaminating samples prior to culture is inherently detrimental to mycobacterial viability. As a result, the overall sensitivity of cultures is only 80–85% compared to a composite reference standard,[6] but significantly lower in clinical conditions where the bacterial load in sputum is low (i.e. paucibacillary TB disease) such as certain patients with HIV-infection,[13] children,[14] and extra-pulmonary TB.[15] Other individuals with active disease harboring non-culturable organisms in sputum include subjects with unstable latent TB infection and early sub-clinical disease that have “percolating” organisms,[16] and those with old untreated TB.[17, 18] In addition, “persistent” organisms after antituberculous therapy may represent the paucibacillary TB pool for poor treatment outcomes.[17] Without culture confirmation, paucibacillary TB is rarely identified leading to empirical treatment, over- or underdiagnosis, and increased morbidity and mortality.[19]

We have developed the “Totally Optimized PCR (TOP) TB assay”, a new nucleic acid amplification test (NAAT) that utilizes a combination of efficient sample processing, novel gene target selection, modern primer design techniques, and an extended PCR for selective target isolation and amplification. The assay is highly specific for Mycobacteria in the MTB complex and therefore is not affected by background genomic noise, which enables detection with heightened sensitivity. We report here results of *in silico* and *in vitro* data. To compare accuracy of the TOP TB assay with culture and the leading molecular test (e.g. Xpert MTB/RIF), we then performed a cross-sectional evaluation using specimens from HIV-infected subjects enrolled into an existing prospective diagnostic study in Uganda.

## Materials and Methods

Ethical approvals: The studies were approved by the Institutional Review Boards at Boston University Medical Center, Mbarara University of Science and Technology, and the Uganda National Council for Science and Technology. Samples were shipped to Boston under a Material Transfer Agreement for DNA sequencing.

### TOP TB assay

The assay targets a gene (*ponA1*) involved in the assembly of peptidoglycans in the MTB bacterial wall.[20] The assay’s diagnostic primer set (3-ponA-F/R) targets sequences unique to all species in the MTB complex (Section IA, Fig A and Table A in S1 File). Amplicons generated by 3-ponA were detected using a capture-probe colorimetric assay, and the resultant Optical Densities (OD) provided a semi-quantitative measurement of MTB bacillary load.[21] A more detailed description of the TOP TB assay and its associated laboratory methods, including sample processing and DNA extraction, PCR amplification and amplicon detection are provided in S1 File (Sections IA and IB).

### PCR genotyping

To establish the presence of MTB DNA, we tested all specimens with primer set 2-ponA-F/Ra (used for genotyping), which targets a section of *ponA1* that is sufficiently distant (~1,100 bp) from the 3-ponA target (used for diagnosis) to remain unaffected by amplicons generated with primer 3-ponA (Section IC, Fig A and Table A in S1 File). 2-ponA PCR products were sequenced to distinguish among five possible genetic variants of MTB (genotypes 0^T^, 1^T^, 2, 3 and 4) (Section IC in S1 File). The sequencing nomenclature (Fig B in S1 File) and the genetic correspondence of 2-ponA genotypes to other familiar MTB whole genome genotyping methods are shown in the Appendix (Fig C and Table B in S1 File).

### Clinical study

After completing pre-clinical studies, we tested a convenience sample of discarded sputum specimens obtained from participants enrolled into a cross-sectional TB diagnostic study in Uganda. Table C in S1 File summarizes: the study design, a description of the subjects, and methodology (including reference methods).

#### Setting

The study was conducted at the Epicentre/ Médecins sans Frontières Laboratory located at Mbarara University of Science and Technology in Mbarara, Uganda. With an estimated TB incidence of 166 cases per 100,000 inhabitants, Uganda is on the WHO list of high burden TB countries; the prevalence of HIV infection among TB patients is 48%.[1] Mbarara District is situated in the South Western (SW) zone of the Uganda National Tuberculosis and Leprosy Programme (NTLP). According to NTLP laboratory activity reports, 8,423 TB patients were registered in the SW zone (incidence rate 290 per 100,000). Of the 3701 TB suspects from Mbarara District, 668 (18%) were AFB smear-positive, and 68% were HIV infected.[22]

#### Study population

Participants for this study were enrolled into a prospective cross-sectional study designed to independently evaluate the diagnostic accuracy of a new AFB smear microscopy method [23] and Xpert MTB/RIF, with liquid media culture (manual MGIT 960) as the reference method.[24] From September 4^th^ 2012 to April 11^th^ 2014, the parent study enrolled 1,047 (737 HIV-infected and 310 HIV-uninfected) consecutive TB suspects admitted to the wards or attending any of the outpatient clinics of the Mbarara Regional Referral Hospital or the Municipality Health Centre in Mbarara city. Eligible participants were adult (≥18 years), TB suspects (≥2 weeks of cough + at least one other symptoms of TB) [25] willing to follow the study protocol. Patients were excluded if they had received antituberculous drugs within three days, were too ill to consent, or presented with disseminated or extra-pulmonary TB without cough.

#### Study design and measurements

Participants had a standardized TB evaluation, HIV testing and provided three spontaneously expectorated (≥2 mL) sputum samples that included one early morning and two spot samples in a 24-hr period. One of the spot samples (selected by randomization) [24] was tested for direct AFB smear, Xpert MTB/RIF and culture.

#### Sample handling prior to TOP processing and testing

Specimens from the last 261 HIV-infected participants enrolled into the parent study were available for this study. TOP testing was done on the discarded portion of a pellet processed for Xpert MTB/RIF. A ~1mL aliquot was frozen at -80°C for two to six months prior to TOP testing; after thawing, the pellet was washed to remove N-acetyl-L-cysteine / sodium hydroxide solution, [26] processed for TOP and tested in a single batch at the Epicentre laboratory in Mbarara. Study personnel were blind to routine TB results; coded results were later linked via a study identification number.

#### Standard Laboratory Methods

The Epicentre/ Médecins sans Frontières Laboratory has quality assurance (QA) and quality control (QC) protocols, and well-trained personnel with extensive experience in laboratory based TB research. The appearance of sputa specimens was classified as purulent, mucopurulent, mucosalivary or, salivary by the microbiology technicians according to international laboratory guidelines.[27] We used the light-emitting diode-auramine fluorescence technique (FluorescenS^®^ LED system, Bergman Labora, Danderyd, Sweden) for direct AFB microscopy on each specimen and reported the results according to the WHO grading scale.[28] The specimen was then decontaminated using the N-acetyl-L-cysteine (0.5%) / sodium hydroxide (1.5%) method.[26] For the reference culture method, we inoculated 500 μl into one manual-testing MGIT 960 (Becton, Dickinson, Franklin Lakes, NJ). We reported a negative culture result after 56 days of incubation at 37°C. Contamination in MGIT media was ruled out using Ziehl-Neelsen (ZN) microscopy and culture on blood agar. For all positive MGIT cultures, we differentiated between *M*. *tuberculosis* and non-tuberculous mycobacteria (NTM) using the SD TB Ag MPT64 Rapid system (SD Bioline, Kyongi-do, South Korea), following the manufacturer’s instructions. The GenoType Mycobacterium CM/AS identification kit (Hain Lifescience, Nehren, Germany) was used for identification of NTM. The Xpert^®^ MTB/RIF assay (Cepheid, Sunnyvale, CA, U.S.A.) was performed according to the manufacturer’s instructions.

### Analytical strategy

We report the results according to the Standards for Reporting of Diagnostic Accuracy (STARD) guidelines.[29, 30] We calculated the diagnostic cut-off for TOP OD using a cut-off value of three standard deviations above the mean of the OD values of negative controls (e.g. laboratory cut-off). [31] As a sensitivity analysis, we used receiver operating curve (ROC) tools to determine the cut-off that simultaneously maximized sensitivity and specificity (e.g. ROC cut-off). We estimated per-patient sensitivity and specificity using culture as the reference standard, using all available results to adjudicate TB status. We also estimated per-patient sensitivity and specificity using a Composite Reference Standard (CRS) that included culture, MTB sequencing (e.g. 2-ponA genotyping), a NAAT other than TOP (e.g. Xpert MTB/RIF) and AFB smear, as described. [15, 32–34] We analyzed patient characteristics according to TOP and culture results using Kruskal-Wallis (for continuous data) and Fisher’s exact test (for categorical data), and compared groups using Wilcoxon and Fisher’s exact tests. Variables with p< 0.1 and those considered to be clinically significant were included in multivariate logistic and ordinal logistic regression models. In the former we consider correlates with culture+/TOP+ compared to culture-/TOP+ individuals. In the latter, we compare all three outcomes ordinally. For these models we group X-ray results into two categories: Normal/Minimal versus Moderate/Far Advanced. The models controlled for age, sex, previous TB treatment, sputum appearance, sputum volume (only for first model), and X-ray (2 category).

## Results

### Preclinical studies

In the preclinical phase, TOP TB’s primer set 3-ponA (used for diagnosis) demonstrated: i) excellent analytical sensitivity when clinical sputum samples were “spiked” with *Mycobacterium bovis* Bacille Calmette-Guérin (Fig D, top in S1 File); ii) semi-quantitative detection capability over a range of MTB loads (Fig D, bottom in S1 File); iii) high analytical specificity, testing negative against a panel of 18 common respiratory bacteria and other microorganisms (Fig E in S1 File), and; iv) high specificity against non-tuberculous mycobacteria (Fig F in S1 File). The 2-ponA primer set (used for sequencing) demonstrated a ~8–10% lower analytical sensitivity but similar analytical specificity in the preclinical phase of testing (data not shown).

### Clinical study

**Fig 1:**
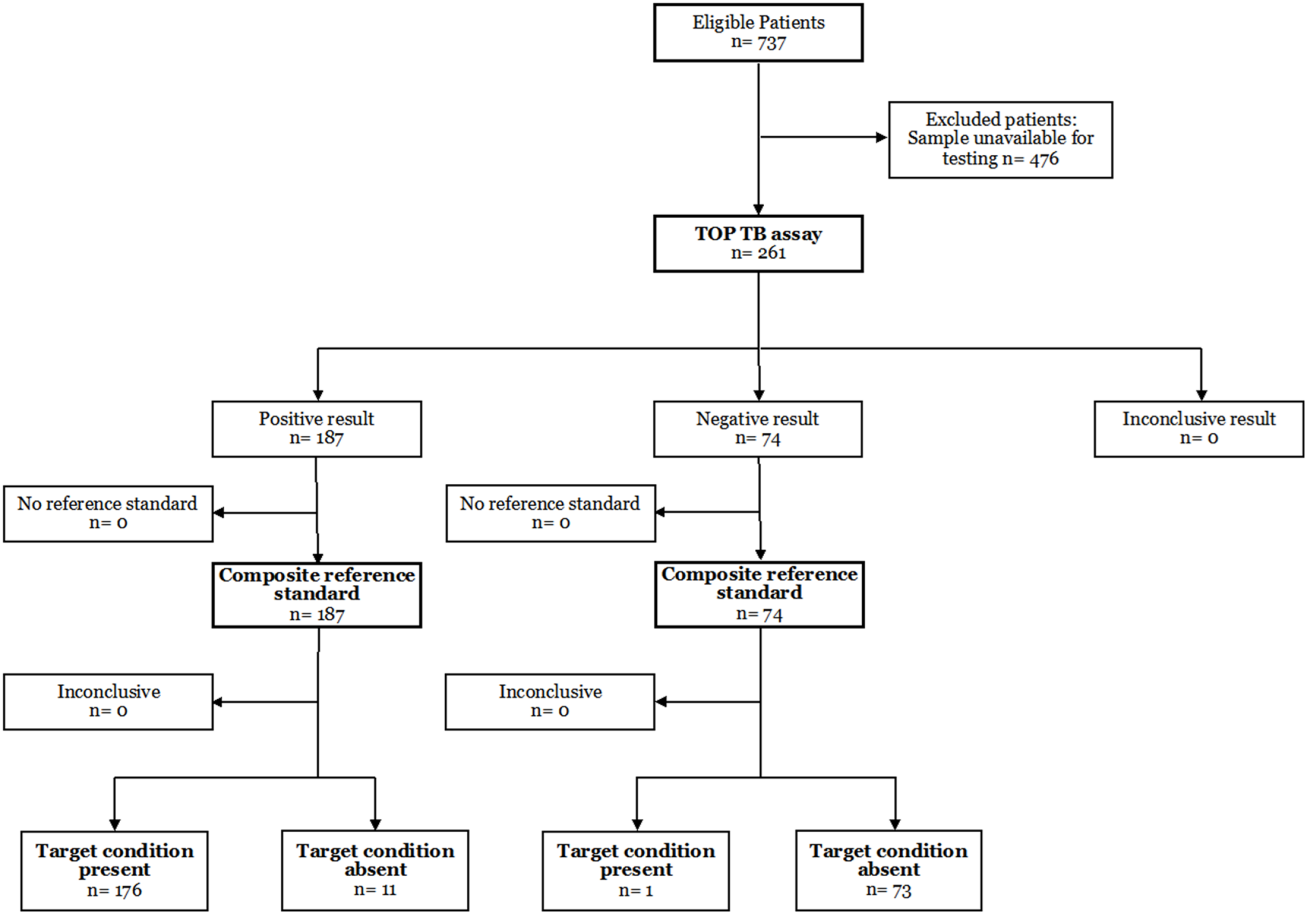
Study profile.

We then evaluated the TOP TB assay in 261 HIV-infected pulmonary TB suspects enrolled into the parent study between October 2, 2013 and April 11, 2014 (Fig 1). Table 1 shows characteristics of the study cohort according to culture and TOP TB assay results.

**Table 1:** Characteristics of 261 HIV-infected pulmonary tuberculosis suspects in Mbarara, Uganda by *M*. *tuberculosis* culture and TOP TB assay results.

Characteristic	Overall	Culture positive	Culture negative		P value	
		TOP positive	TOP positive	TOP negative	Overall^1^	Two-way^2^
N	261	48	139	74		
Age (years)	39.0 [30.5–47.0]	33.5 [28.0–40.0]	42.0 [33.0–49.0]	38.5 [31.0–48.8]	0.002	<0.001
Female sex	138 (53)	12 (25)	78 (56)	48 (65)	<0.001	<0.001
Previous TB treatment*	33 (13)	3 (6)	23 (17)	7 (9)	0.04+	0.05+
Years since previous TB treatment	9.3 [5.9–10.9]	5.4	9.3 [5.9–10.9]	11.9 [10.3–12.0]	0.28#	0.16#
	N = 13	N = 1	N = 9	N = 3		
CD4 (cells/mL)*	322 [104–495]	182 [54–338]	343.5 [93–457]	355 [158–590]	0.06	0.13
	N = 172	N = 22	N = 92	N = 58		
Sputum volume (mL)	3 [2–5]	4 [3–5]	3 [2–4.5]	3 [2–5]	0.33#	0.16#
Sputum appearance^					<0.001+	0.05+
Purulent	69 (27)	20 (42)	36 (26)	13 (18)		
Mucoid	36 (13)	10 (25)	23 (17)	3 (4)		
Salivary	156 (60)	18 (33)	80 (58)	58 (78)		
Chest radiograph*					0.04+	0.05+
Normal	29/103 (28)	2/17 (12)	14/47 (30)	13/39 (33)		
Minimal	17/103 (17)	1/17 (6)	8/47 (17)	8/39 (21)		
Moderate	46/103 (45)	8/17 (47)	21/47 (45)	17/39 (44)		
Far advanced	11/103 (11)	6/17 (35)	4/47 (9)	1/39 (3)		
Cavitation present	18/104 (17)	5/17 (29)	7/48 (15)	6/39 (15)	0.37+	0.27+
Sputum AFB smear*					−	<0.001+
Negative	222 (85)	11 (23)	137 (99)	74 (100)		
Scanty	9 (3)	8 (17)	1 (1)	0 (0)		
1+	10 (4)	10 (21)	0 (0)	0 (0)		
2+	7 (3)	6 (13)	1 (1)	0 (0)		
3+	13 (5)	13 (27)	0 (0)	0 (0)		
Sputum MGIT culture						
Positive	48 (18)	48 (100)	0/139 (0)	0/74 (0)	-	-
Contaminated	12 (5)	−	2/139 (1)	10/74 (14)		
MGIT DTP (days)	19 [13–33]	19 [13–33]	NA	NA	-	
Xpert Mtb/RIF *						
Positive	50/259 (19)	45/47 (96)	3/139 (2)	2/73 (3)	<0.001+	<0.001+
Indeterminate	4/259 (2)	1/47 (2)	1/139 (1)	2/73 (3)		
*M*. *tuberculosis* 2-ponA genotype						
0^T^	5 (2)	4 (8)	1 (1)	0	-	0.005+
1^T^	6 (2)	3 (6)	3 (2)	0		
1	2 (1)	0	2 (1)	0		
2	40 (15)	16 (33)	24 (17)	0		
3	93 (36)	18 (38)	74 (53)	1 (1)		
4	27 (10)	3 (6)	24 (17)	0		
Neg	88 (34)	4 (8)	11 (8)	73 (99)		

Values are median [interquartile range] or number (percentage), unless otherwise specified

MGIT = Mycobacterial Growth Indicator Index (BACTEC 960, Becton Dickinson, U.S.A.); DTP = Days-to-positive; AFB = Acid-fast bacilli

^1^ P overall = Comparison between three groups

^2^ P two-way = Comparison between culture-positive/ TOP-positive vs. culture-negative/ TOP-positive

* Missing information: Previous TB treatment (1); CD4 cell count (85); Time since previous TB treatment (20); Chest X-ray extent of disease (154), cavitation (153); Sputum AFB smear (1); Xpert MTB/RIF (2)

^ Purulent sputum category includes purulent and muco-purulent; Mucoid category includes mucoid and muco-salivary

^+^ Fisher’s exact test.

^#^ Kruskal-Wallis test or Wilcoxon test.

**Fig 2:**
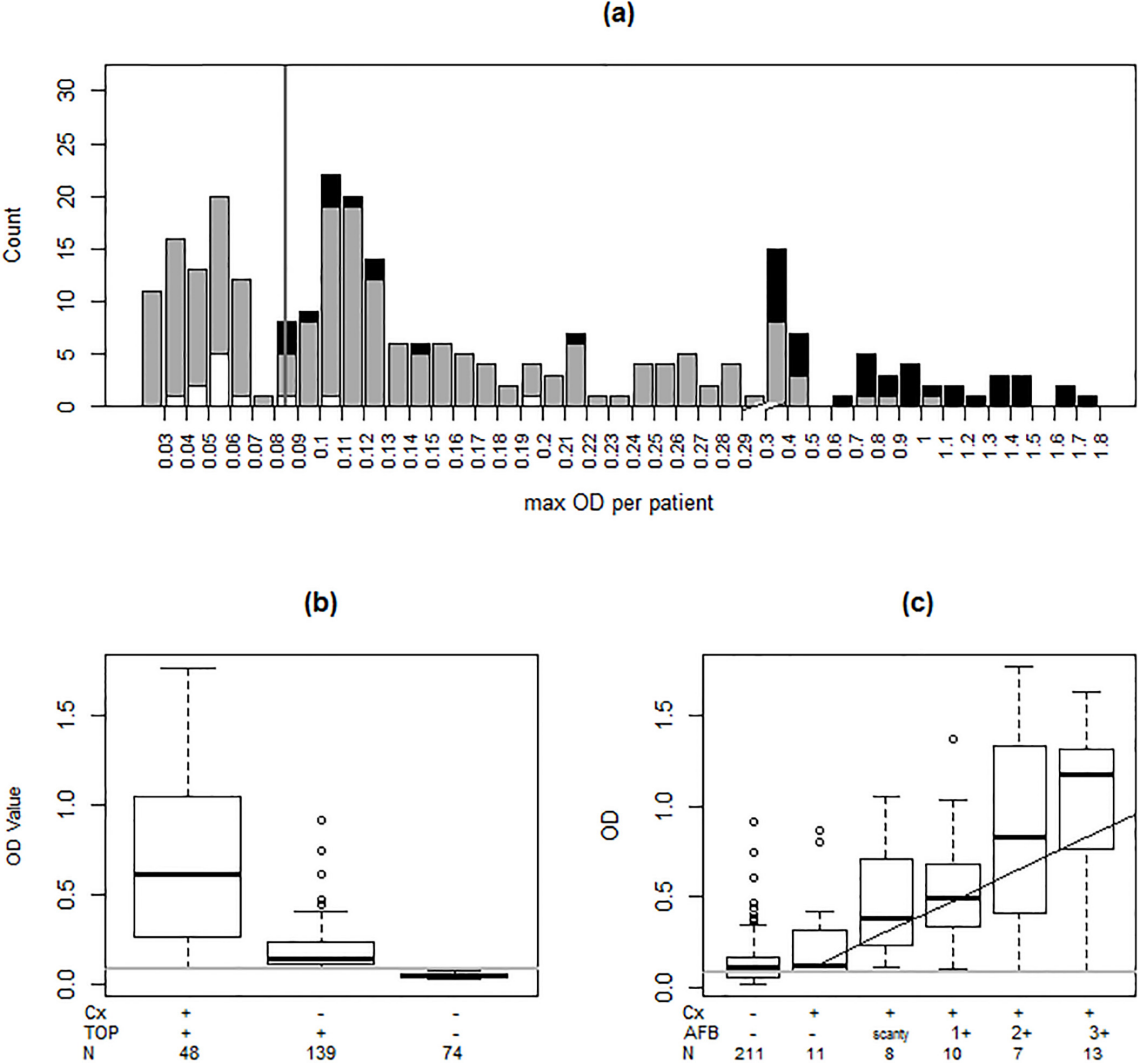
TOP TB assay results in 261 HIV-infected TB suspects from Mbarara, Uganda. **(a)** Vertical line denotes the laboratory cut-off TOP OD (0.0854) for a positive test. Histograms represent the number of subjects with culture-positive (black), culture-contaminated (white) and culture-negative (grey) results, by TOP OD values. The X-axis is zoomed-in at the lower end of TOP OD values (0.100 to 0.300) to show the large number of subjects in this section of the graph. **(b)** Group TOP OD values according to culture (Cx) and TOP results (group means are 0.67, 0.19, and 0.05, from left to right). The mean TOP OD of culture-positive/TOP-positive (0.67) samples was higher than in culture-negative/TOP-positive (0.19, P<0.0001), suggesting a low bacterial load content in many HIV-infected TB suspects. **(c)** Median TOP ODs paralleled sputum AFB grades (P<0.0001), demonstrating the semi-quantitative performance of the TOP TB assay. One subject with a scanty AFB reading and a contaminated culture was excluded (TOP OD 0.103). A smoothing spline fit to the data is shown.

As shown in Fig 2, 48/261 (18%) patients were culture-positive, all of which were also TOP-positive. Seventy-four (28%) were culture-negative (N = 64) or contaminated (N = 10) and were TOP-negative; 139 (53%) were culture-negative (N = 137) or contaminated (N = 2) but TOP-positive (Fig 2a). The distribution of TOP ODs by culture and AFB smear are shown in Fig 2b and 2c, respectively. Of the 139 culture-negative/ TOP-positive samples, 2-ponA sequencing confirmed the presence of MTB DNA in 128 (92%). The sensitivity and specificity of TOP and Xpert MTB/RIF compared to culture or a CRS are shown in Table 2; the breakdown of results included in the CRS is shown in Table D in S1 File. We were unable to sequence MTB from 11/139 (8%) culture-negative/ TOP-positive specimens with low TOP OD values (median 0.13, IQR 0.11–0.34).

**Table 2:** Per-patient sensitivity and specificity of the TOP TB assay, Xpert MTB/RIF and culture in 261 HIV-infected tuberculosis suspects according to a reference standard established by *M*. *tuberculosis* culture or a Composite Reference Standard (CRS) in Mbarara, Uganda.

Diagnostic Method	MTB detected (N)	MTB not detected (N)	Sensitivity		Specificity		PPV		NPV	
			n/N	% (95% CI)	n/N	% (95% CI)	n/N	% (95% CI)	n/N	% (95% CI)
	**48**	**213**^1^	**Culture reference standard**							
Xpert MTB/RIF ^2^	50	209	45/47	96% (84, 99)	207/212	98% (94, 99)	45/50	90% (77, 96)	207/209	99% (96, 100)
TOP TB assay	187	74	48/48	100% (93, 100)	74/213	35% (28, 41)	48/137	26% (20, 33)	74/74	100% (95, 100)
	**177**	**84**	**Composite reference standard** ^3^							
Culture	48	213	48/177	27% (21, 34)	84/84	100% (96, 100)	48/48	100% (93, 100)	84/211	40% (33, 47)
Xpert MTB/RIF	50	209	48/176	27% (21, 35)	81/83	98% (91, 100)	48/50	96% (85, 99)	81/209	39% (32, 46)
TOP TB assay	187	74	176/177	99% (97, 100)	73/84	87% (77, 93)	176/187	94% (89, 97)	73/74	99% (93, 100)

Definition of abbreviations: CI = Confidence interval; CRS = Composite reference standard; MTB = *Mycobacterium tuberculosis*; NPV = Negative predictive value; PPV = Positive predictive value

^1^ Includes 12 patients with contaminated culture results

^2^ Two Xpert MTB/RIF results were missing and 4 had indeterminate result (N = 259)

^3^ Composite Reference Standard (CRS) included *M*. *tuberculosis* culture, *M*. *tuberculosis* sequencing (e.g. 2-ponA genotyping), a NAAT other than TOP (e.g. Xpert MTB/RIF), and AFB smear.[15] The breakdown of CRS results is shown in Table S4 (Appendix)

In univariate analyses that compared culture-positive/TOP-positive vs. culture-negative/ TOP-positive patients (Table 1), the latter were more likely to be older (P<0·001); women (P<0·001); have a salivary sputum (P = 0·05); have a previous history of TB disease (P = 0·05), and have early TB disease as measured by sputum AFB smear grade (P<0·001) and chest radiograph (P = 0·05). In a multivariate analysis comparing culture+/TOP+ to culture-/TOP+ patients, age (p = 0.003) and gender (p = 0.002) remained statistically significant. In a comparison of all three TOP/culture categories from Table 1, multivariate results revealed that age (p = 0.02) and gender (p<0.001) were statistically significant and previous TB treatment was marginally significant (p = 0.10).

The cut-off for TOP OD values was determined to be 0.0854 using the laboratory criterion (e.g. +/- three standard deviations criterion). When we used ROC analysis with 100 random observations, we found the cut-off to be 0.088 leading to reclassification of only 4 individuals (Table E in S1 File). The area under the ROC curve was 0.86 for culture and 0.95 for the CRS using TOP OD as the diagnostic test (Fig G in S1 File).

### *M*. *tuberculosis* sequencing results

**Fig 3:**
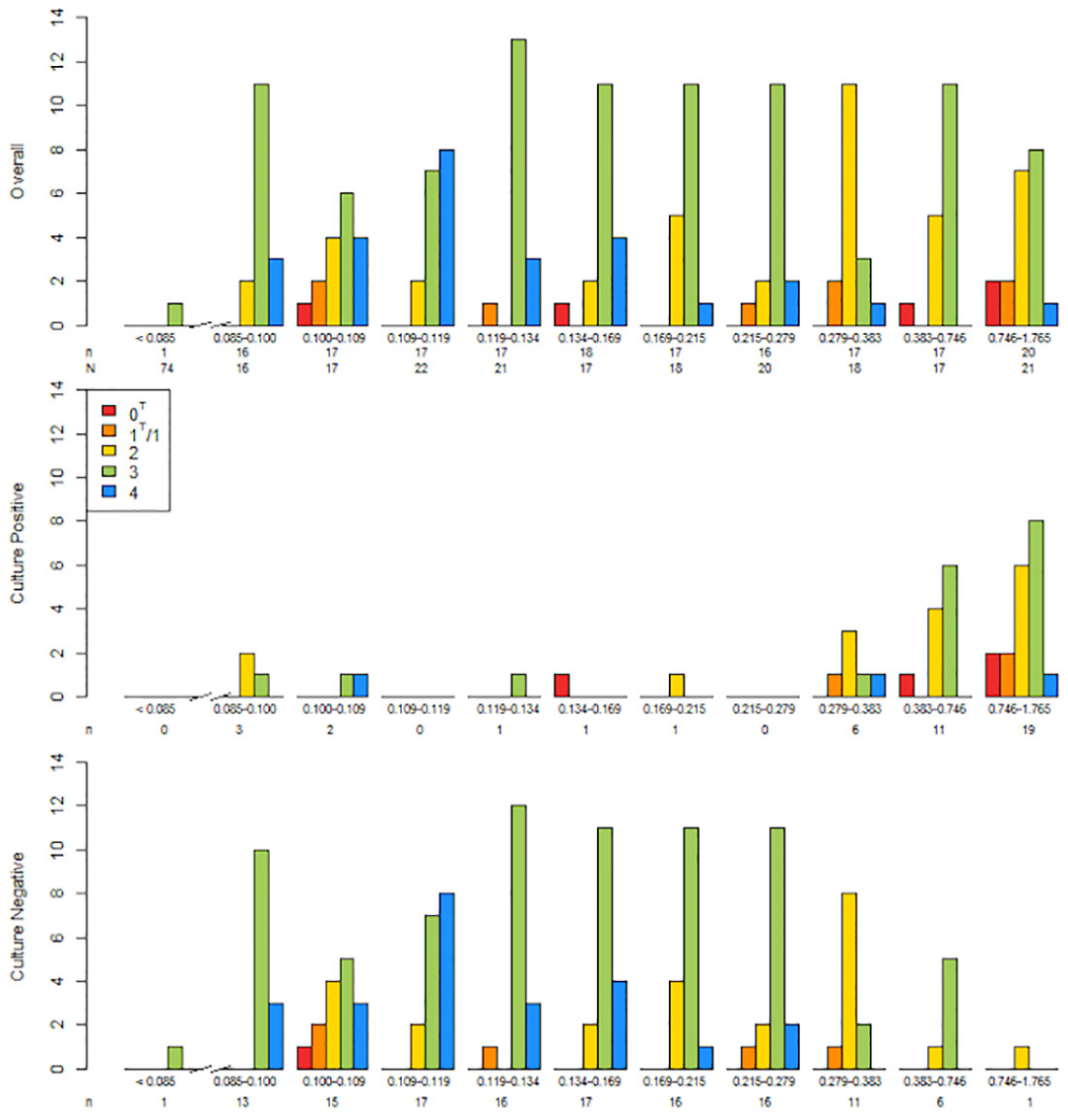
Distribution of 2-ponA genotypes (sequencing results) in 261 HIV-infected pulmonary TB suspects in Mbarara, Uganda according to TOP TB assay OD values. Results are shown for all subjects (top), and then separated by those that were culture-positive (middle) and culture-negative (bottom). The X-axis is divided into groups of subjects with similar bacterial loads as measured by TOP OD values. The first group (far left) includes 74 subjects that were culture-negative and TOP-negative (3-ponA primer). N denotes the number of subjects in each TOP OD group; n denotes the number of subjects with a positive 2-ponA genotype in each group. A 2-ponA genotype could not be identified in 8% (4/48) culture-positive/TOP-positive samples and 8% (11/139) culture-negative/TOP-positive samples. A 2-ponA genotype was identified in 1% (1/74) of culture-negative/TOP-negative samples.

**Fig 4:**
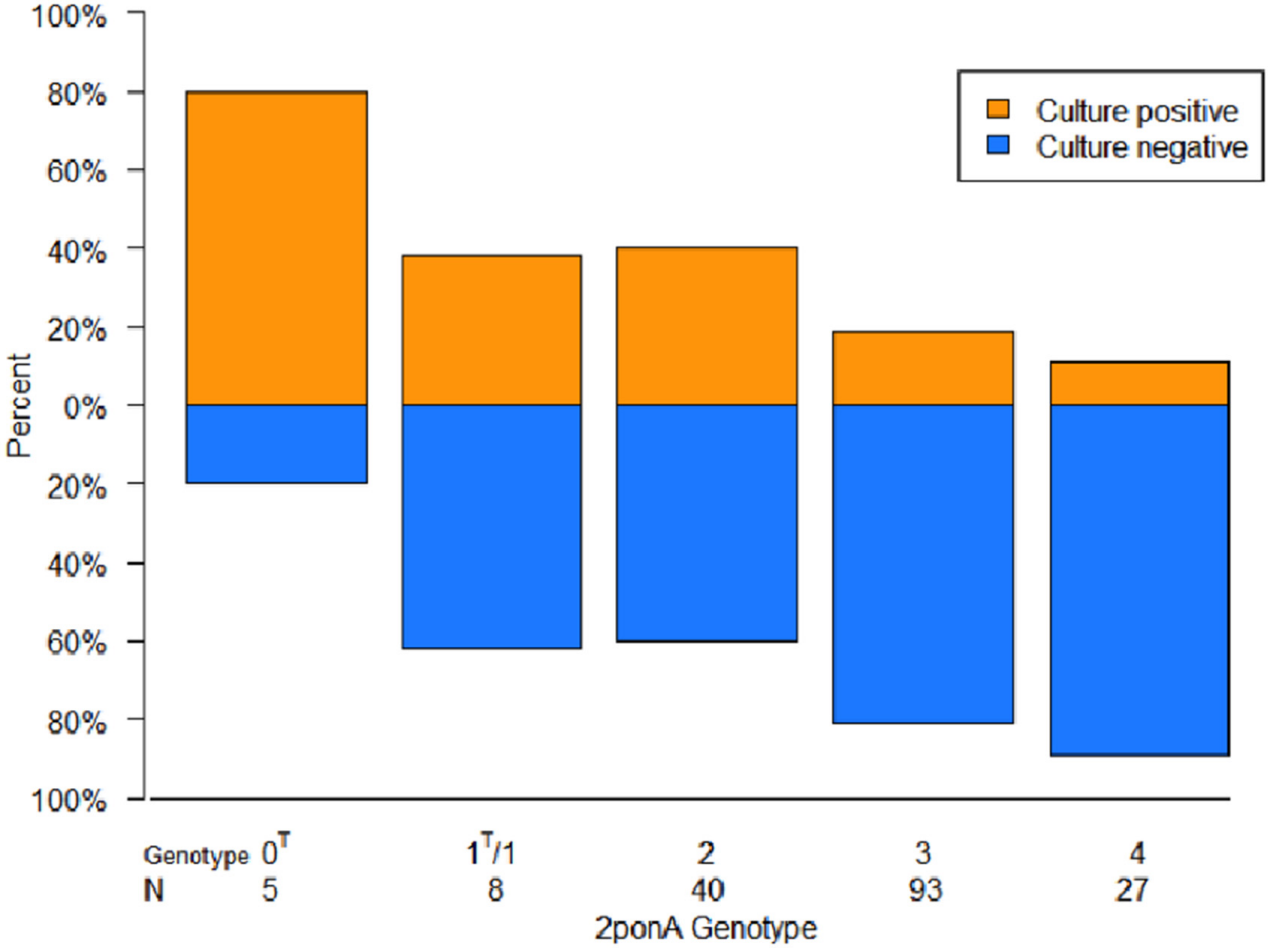
Distribution of 2-ponA genotypes by culture results. Each histogram represents 100% of strains for each 2-ponA genotype. N denotes the number of strains in each group. The proportion of samples that were culture-positive decreased with the number of Proline codon deletions (e.g. Genotype 4 = 4 Proline deletions) in the poly-Proline track in the *ponA1* region targeted by the 2-ponA primer (see Fig B in S1 File) (P = 0.002).

The relative frequency and distribution of 2-ponA genotypes differed significantly according to TOP OD values (Fig 3 and Table 2; P = 0·005); in particular, genotype 4 strains were mostly restricted to culture-negative samples with low ODs (Fig 3). As shown in Fig 4, 2-ponA genotypes had variable growth in culture (P = 0·002).

## Discussion

Our study provides strong evidence that the TOP TB assay accurately detects trace amounts of MTB DNA in the sputum of HIV-infected TB suspects who otherwise may yield a negative culture. Currently a culture diagnosis is the optimal reference standard for diagnosis. In the absence of culture to determine specificity, we established the validity of positive diagnosis using a composite reference standard and, most importantly, we sequenced MTB from culture-negative specimens. We used a reproducible genotyping method that is supported by the genetic signature of a global collection of MTB clinical isolates representing all major phylogenetic lineages.

The natural history of pulmonary TB in HIV-uninfected adults is traditionally viewed as a sub-acute or chronic illness whose progression is accompanied by increasing bacterial loads in sputum, paralleling worsening disease severity on chest radiography.[4, 6] The paradigm states that patients with early TB are often smear- and culture-negative, and with an increase in severity, most patients become smear-negative/culture-positive first, and eventually, smear- and culture-positive. However, a variety of epidemiologic studies including household contact investigations, molecular epidemiology and TB screening studies have demonstrated that the rate of disease progression in humans can be highly variable, perhaps as a consequence of low or stagnant MTB bacterial loads in sputum.[4, 35, 36] Furthermore, effective diagnostic and treatment programs that seek out cases to identify patients with most advanced disease (i.e. AFB smear- and culture-positive) may produce an epidemiological shift that results in the remaining populations with suspected TB of having a higher prevalence of early TB disease (i.e. smear-negative/culture-negative), that are the most difficult to confirm bacteriologically.[4] For example, in the U.S. during the 1980s, 90% of TB cases were confirmed by culture but this proportion decreased to 77% by 2013; [37] in some settings (e.g. Boston, MA and Alberta, Canada), ~50% of notified TB cases are culture-negative.[38, 39] Therefore, culture-negative TB disease is a global problem resulting from both biological and epidemiological factors, for which there are currently limited solutions beyond the initiation of empirical antituberculous treatment based on clinical algorithms.[40]

TOP TB enables enhanced detection of MTB in the sputum of TB suspects with HIV/AIDS, perhaps one of the largest and most vulnerable (together with children) populations with paucibacillary TB disease. In the early phase of assay development, our *in vitro* results suggested an analytical sensitivity of 1–4 colony-forming units (CFU) of MTB per mL, a level of detection greater than culture (e.g. 10–100 CFU/ml). With nullification of culture as the reference method, we anticipated challenges in validating the accuracy of our results. Several analytical methods have been recommended when dealing with imperfect reference methods such as mycobacterial cultures.[15, 32, 33, 41] The use of one of these—“discrepant analysis”, or the use of a third test, is limiting because usually it is not applied consistently across all the specimens that are being examined, only the ones where the new test result conflicts with the “gold standard”.[32, 33, 41] Our methods minimize the limitations of using discrepant analysis to evaluate NAATs because we tested all specimens, and because of the complete lack of overlap (lack of ‘dependence’) between the 3-ponA (diagnostic) and 2-ponA (genotyping) primers. Furthermore, the use of both clinical and epidemiologic data to act further as a referee, the latter demonstrating variability of genotypes according to TOP ODs add strength to the interpretation of a positive TOP in the face of a negative culture. Our results may be novel in the TB diagnostic field but the development of molecular tests with sensitivity superior to culture is not new in clinical settings.[42] Based on our results, the inclusion of 2-ponA genotypes into a composite reference standard to evaluate the performance of TOP follows standard practices in the TB diagnostic field [5, 15, 34], and beyond. [43] Importantly, because the sensitivity of the primer used for sequencing is ~8–10% lower that the diagnostic primer, 11/139 (8%) culture-negative/ TOP-positive specimens were adjudicated as false-positive TOP results, lowering the specificity of the assay in this study. In other ongoing studies with non-HIV sputum samples, the specificity of TOP has been 93% to 100% (data not shown).

Our results suggest that the TOP assay is sufficiently sensitive to overcome well-recognized difficulties with sputum procurement, such as inadequate specimen volumes and/or poor quality of specimens (e.g. excess saliva)–a problem that is thought to diminish diagnosis of TB in women disproportionately.[44–47] This also raises the possibility of using sputum to diagnose paucibacillary TB when non-pulmonary clinical specimens (blood, gastric aspirates, urine, stool) have otherwise been thought necessary to establish the diagnosis.[5, 12, 48] Interestingly, our sequencing data establish a potential link between diagnosis, epidemiology and pathogenic behavior of MTB in humans. In particular, a high frequency of genotype 4 MTB isolates was noted in HIV-infected patients with low TOP ODs in Uganda. We had rarely observed this variant in the global collection of clinical isolates before these studies were started, raising the possibility that genotype 4 strains are uniquely adapted to HIV-infected hosts and cause predominantly culture-negative TB disease.

Our study has limitations. Our results were obtained by testing a convenience sample of low-volume, discarded, stored sputum specimens from an existing diagnostic clinical study; therefore, performance of the TOP assay may have been underestimated. The selection of the study population was solely based on when the TOP TB assay was ready for clinical testing (October 2013) rather than selection bias, as shown by the results of the parent study that included the entire population of HIV-infected patients. [24] The lack of clinical follow-up of subjects limits the clinical interpretation of certain results. For example, TOP was positive in numerous patients with a current or past history of treatment for TB, which complicated the clinical interpretation of negative cultures; interestingly, a similar phenomenon has recently been described with Xpert MTB/RIF, although a discrepant analysis was not performed.[49, 50] A positive TOP result likely represented either residual (dead) MTB DNA, viable but non-culturable organisms or bacterial persistence after treatment, the latter a potential harbinger of TB recurrence and/or risk of drug resistance.[17, 51, 52] Admittedly, detection of trace amounts of MTB DNA may be due to bacterial “spillage” from a dormant lung foci or low level bacterial replication that may not require treatment. The colorimetric readout uses a study-specific cut-off value to establish the “Limit of Blank”, a key assay parameter.[31] Finally, in its current embodiment, the TOP TB assay does not include provisions for detecting drug-resistant TB. However, the primary global need is for a rapid and reliable triage test.[5, 12]

## Conclusions

Culture-negative TB is widespread, resulting from several biologic and epidemiologic factors. By shifting diagnostic emphasis to early detection, the TOP TB assay broadens sensitive and accurate detection of MTB across the entire clinical spectrum of TB disease. Our findings will require validation with clinical outcomes obtained prospectively. If the operating characteristics of the TOP assay are confirmed in future studies, it would be justified as a triage or “TB rule out” test.

## Supporting Information

S1 File Supplementary Appendix. **Experimental Laboratory methods**
Technical aspects of TOP TB assayTOP TB assay laboratory methods***M*. *tuberculosis* sequencing and genotype nomenclature****Supporting Figures**
Fig A: DNA sequence alignment of *M*. *tuberculosis ponA1*Fig B: Sequence representation of five possible 2-PonA genotypes of *M*. *tuberculosis* clinical isolatesFig C: Genetic correspondence of *M*. *tuberculosis* 2-ponA genotypes with other common whole-genome genotyping methodsFig D: Analytical sensitivity of 3-ponA primerFig E: Analytical specificity of 3-ponA primerFig F: Specificity of TOP TB assay against non-tuberculous mycobacteriaFig G: Receiver operating curve (ROC) analysis of TOP TB assay results according to culture or composite reference standard**Supporting Tables**
Table A: Description of 3-ponA and 2-ponA primersTable B: Genetic correspondence of *M*. *tuberculosis* 2-ponA genotypes with other common whole-genome genotyping methodsTable C: Summary of Uganda clinical studyTable D: Breakdown of results for the Composite Reference StandardTable E: Sensitivity analysis using TOP TB assay cut-off determined by ROC analysis**References**
(DOC)
