# Supplementary material for: Detection and Quantification of Mycobacterium tuberculosis in the Sputum of Culture-Negative HIV-infected Pulmonary Tuberculosis Suspects: A Proof-of-Concept Study
Source: PLoS One. 2016 Jul 8;11(7):e0158371. doi: 10.1371/journal.pone.0158371 (PMC4938528; doi:10.1371/journal.pone.0158371)
Supplement: S1 File — Experimental Laboratory methods Technical aspects of TOP TB assayTOP TB assay laboratory methodsM. tuberculosis sequencing and genotype nomenclatureSupporting Figures Fig A: DNA sequence alignment of M. tuberculosis ponA1Fig B: Sequence representation of five possible 2-PonA genotypes of M. tuberculosis clinical isolatesFig C: Genetic correspondence of M. tuberculosis 2-ponA genotypes with other common whole-genome genotyping methodsFig D: Analytical sensitivity of 3-ponA primerFig E: Analytical specificity of 3-ponA primerFig F: Specificity of TOP TB assay against non-tuberculous mycobacteriaFig G: Receiver operating curve (ROC) analysis of TOP TB assay results according to culture or composite reference standardSupporting Tables Table A: Description of 3-ponA and 2-ponA primersTable B: Genetic correspondence of M. tuberculosis 2-ponA genotypes with other common whole-genome genotyping methodsTable C: Summary of Uganda clinical studyTable D: Breakdown of results for the Composite Reference StandardTable E: Sensitivity analysis using TOP TB assay cut-off determined by ROC analysisReferences (DOC) [file pone.0158371.s001.doc]

**Supplementary Appendix**

**TABLE OF CONTENTS:**

1. **Experimental Laboratory methods**
2. Technical aspects of TOP TB assay
3. TOP TB assay laboratory methods
4. ***M. tuberculosis* sequencing and genotype nomenclature**
5. **Supporting Figures**
   - Figure A: DNA sequence alignment of *M. tuberculosis ponA1*
     - Figure B: Sequence representation of five possible 2-PonA genotypes of *M. tuberculosis* clinical isolates
     - Figure C: Genetic correspondence of *M. tuberculosis* 2-ponA genotypes with other common whole-genome genotyping methods
     - Figure D: Analytical sensitivity of 3-ponA primer
     - Figure E: Analytical specificity of 3-ponA primer
     - Figure F: Specificity of TOP TB assay against non-tuberculous mycobacteria
     - Figure G: Receiver operating curve (ROC) analysis of TOP TB assay results according to culture or composite reference standard
6. **Supporting Tables**
   - - Table A: Description of 3-ponA and 2-ponA primers
     - Table B: Genetic correspondence of *M. tuberculosis* 2-ponA genotypes with other common whole-genome genotyping methods
     - Table C: Summary of Uganda clinical study
     - Table D: Breakdown of results for the Composite Reference Standard
     - Table E: Sensitivity analysis using TOP TB assay cut-off determined by ROC analysis
7. **References**
8. **Experimental Laboratory Methods.**
9. **Technical aspects of TOP TB assay.**

The TOP TB assay is composed of three key features:

1) *Gene target*. We used a hypothesis driven approach to characterize a family of genes that are unique to *M. tuberculosis*. These genes code for proteins involved in the synthesis of peptide-glycan, a key component of the *M. tuberculosis* bacterial wall. The best representative of this gene family *ponA1* provides 100% homology between all mycobacteria in the *M. tuberculosis* complex (*M. tuberculosis, M. africanum, M. bovis, M. microti, M. canetti, M. pinipedii, M. mungi, M. caprae, M. orygis*). The *ponA1* of other mycobacteria and related bacteria are situated at a substantial genetic distance (15%) from *M. tuberculosis* (e.g. Figure A and Figure F)*.*

*2) Primer selection and design*. We designed two novel primer sets that target DNA sequences unique to *M. tuberculosis* in *ponA1* (e.g. Table A and Figure A). The primers meet requirements for efficient amplification of *M. tuberculosis* (e.g. Table A). Primer set 3ponA-F/R (diagnostic primer) amplifies only *M. tuberculosis*, and therefore avoids false-positive signals from other *Mycobacteria* and related species (e.g. Figure A). Primer set 2ponA-F/Ra (genotyping primer) targets informative DNA sequences that distinguish: 1) *M. tuberculosis* from other closely related mycobacteria in the complex, and; 2) between strains of *M. tuberculosis* (see section *Mycobacterial sequencing and genotyping below*). 2-ponA amplicons are sequenced by available commercial methods. Because there is no overlap between the two primer sets (e.g. Table A), they each provide independent confirmation for one another.

*3) PCR amplification*. The PCR method for *M. tuberculosis* detection is an adaptation of a method developed for *C. trachomatis* in clinical samples. The key features of the PCR method employed are: 1) extended thermocycling (60 cycles) tailored for accurate annealing of primers; 2) enhanced amplicon detection with capture probe technology, and 3) semi-quantitative amplification capability. The TOP TB assay PCR platform provides reliable and reproducible detection of target amplicons over a wide range (10^5^ to 1 *M. tuberculosis* genomes/PCR reaction).

1. **TOP TB assay laboratory methods.**

*1). TOP assay sputum processing and DNA extraction*. We use a three-step, color-guided sputum processing method that utilizes the entire sample as follows: i) *Homogenization:* Reagent A (200μl <3 mL specimens and 400 μl for ≥3 mL) is a proprietary solution that contains miniature (0.1 mm) glass beads for efficient cell lysis during vortexing, rapid (<5 min) blood cell break down, chelation of iron, and inhibition of degrading enzymes; ii) *Liquefaction:* Reagent B (200μl <3 mL specimens and 400μl for ≥3 mL) is a proprietary protein denaturation solution with NaOH that is used to liquefy the specimen for ease of pipetting and neutralization, and; iii) *Single vial DNA purification and extraction:* 50μl of the processed sample is placed into 200μl of DNA extraction solution (Epicentre Biotechnologies, Madison, WI, USA) and heated (<5 min) at 52^o^C and then at 97^o^C for degradation of proteins and other macro molecules. Extracted DNA was stored at -80°C until TOP testing to retain optimal DNA quality. We use 10-20 μl of this preparation for PCR testing.

The sample preparation method used has a number of advantages. Homogenization/ liquefaction is performed in the same container the specimen is collected and thus, the vial remains capped through the entire process and storage (minimizing risk of laboratory cross-contamination). The use of low volume reagents (total volume 400-800μl per specimen) has the dual benefit of increasing sensitivity and, circumventing the need to concentrate the specimen by centrifugation, minimizing occupational risk.

2). PCR amplification. Themocycling parameters were performed as described, except for the following changes: 1) PCR was performed using HotStarTaq DNA polymerase (Qiagen); 2) 0.9x PCR buffer was used for the PCR reaction; 3) The annealing temperature for 3-ponA PCR began at 61°C and ended at 56°C for 1 min, and elongation at 72°C for 45 sec. The annealing temperature for 2-ponA PCR began at 61°C and ended at 59°C for 1 min, and elongation at 72°C for 30 sec, and; 4) Primer 3-ponA/F was biotinylated at the 5’ end.

3). Colorimetric detection of amplified of 3-ponA PCR products. PCR products were detected as described. Briefly: 1) PCR products were denatured with 10μL of 3.4% sodium hydroxide; 2) Multi-well plates (Immulon II, Dinex, USA) were coated with the 3-ponA-probe to capture biotinylated PCR products; 3) PCR products were hybridized with 8x sodium saline sulfate EDTA (SSPE) buffer containing 8% formamide, and; 4) Hybridized biotinylated PCR products were detected with ExtrAvidin peroxidase (Sigma, USA) and color was generated using tetramethyl benzidine dihydrochloride (TMB) (BioRad, USA), and absorbance was read at 450 nm in a multi-well plate reader.

4). DNA sequencing of 2-ponA PCR products. After amplification, PCR products were visualized by electrophoresis using 2% agarose gels stained with ethidium bromide. PCR products were excised and purified (Quiagen gel purification) and sent for sequencing (Genewiz, NJ, USA). 2-ponA PCR sequences were aligned with *M. tuberculosis* H37Rv (Y980_0050) to identify deletions and mutations in the poly-Proline track (e.g. Figure B).

1. ***M. tuberculosis* sequencing and genotype nomenclature.**

The gene section targeted by primer set 2ponA-F/Ra contains a poly-proline track (9 consecutive CCG or CCT codons) that is variable among different *M. tuberculosis* strains, a feature that facilitates genotype analysis. Prior to study initiation, we used primer set 2ponA-F/Ra to study a global collection of *M. tuberculosis* (culture-positive) clinical isolates that included: 1) All *M. tuberculosis* genomes that were publically available as of December 2013; 2) Banked isolates (N=155) from two separate studies conducted over an 8 year period in Kampala, Uganda; 3) A set (N=43) of clinically characterized isolates from the NDI strain bank in Vitória, Brazil; and, 4) A set (N=75) of isolates from the Public Health Research Institute (PHRI, Newark, NJ) strain bank that are epidemiologically and genetically well-characterized; these isolates represent a geographically and genetically diverse (all principal phylogenetic groups) group of strains collected over an extended period of time (see Table B). The analysis revealed only five possible genotypes (0^T^, 1^T^, 2, 3 and 4) and sub-variants such as “1” (e.g. Figure B). The genotype nomenclature refers to the number of proline codon deletions in the poly-proline track caused by DNA slippage during bacterial replication (e.g. Figure B). The genetic correspondence of 2ponA genotypes to other familiar *M. tuberculosis* whole-genome typing methods is shown in Figure C and Table B. For this study, we tested all 261 samples to provide a complete distribution of 2-ponA genotypes across TOP OD values.

1. **Supplementary Figures**

Figure A: DNA sequence alignment of *M. tuberculosis* (MTB) *ponA* compared to *M. kansasii* (MKA), *M. avium* (MAV) and *Nocardia sp* (NOC) in the region corresponding to primer set 3-ponA (top) and primer set 2-ponA (bottom). A dot indicates the same nucleotide is shared with MTB*;* a dash denotes that the nucleotide is missing; a nucleotide letter (“A”, “T”,”C”, or “G”) indicates a different base compared to MTB.

**Figure B**: Sequence representation of five possible 2-PonA genotypes of *Mycobacterium tuberculosis* clinical isolates. The region of *ponA1* depicted is a poly-Proline track (n=9) located at the protein C-terminus. Dashes denote the number of missing Proline codons (0, 1, 2, 3 or 4). The superscript “T” (e.g. 0^T^, 1^T^) denotes the presence of a CCT Proline codon at position 5 in the track. Mutation from CCT to CCG at position 5 generates eight identical CCG Proline codons. The single point mutation from T to G at this codon makes the poly-Proline track more genetically unstable and prone to DNA slippage at the time of bacterial replication leading to successive onward Proline codon deletions.


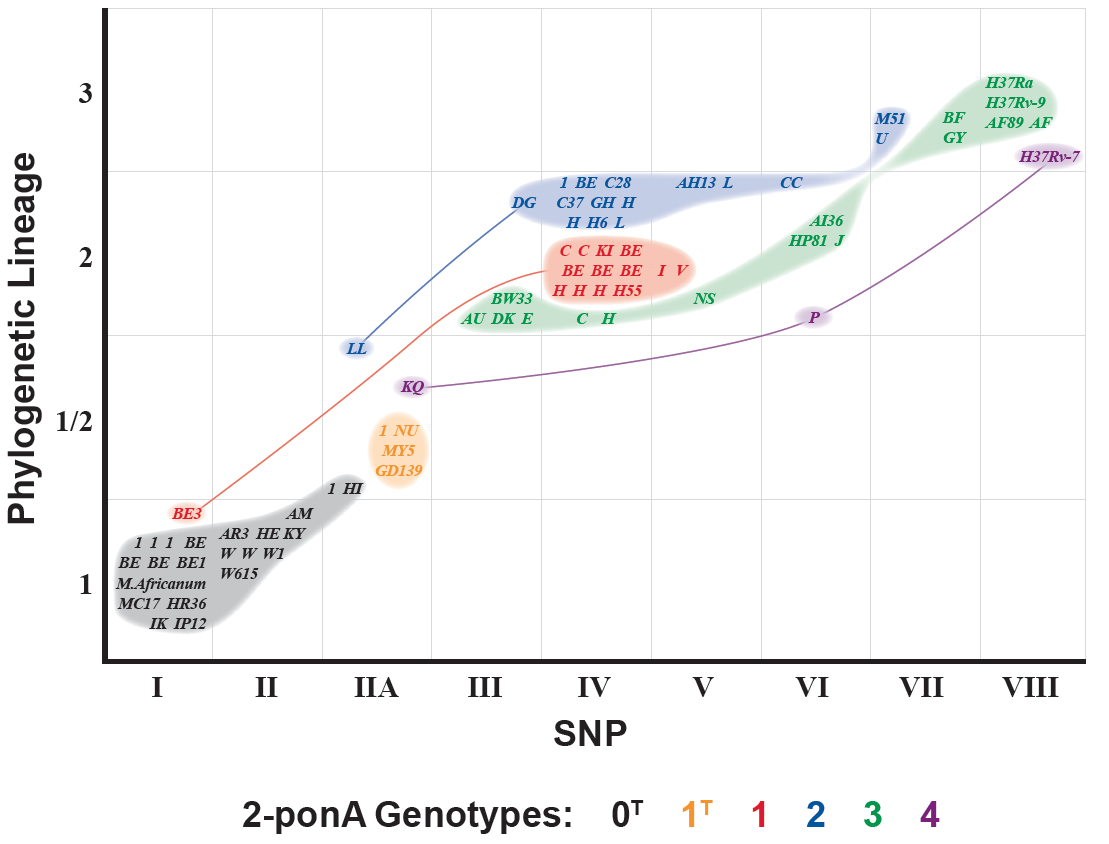


**Figure C**: Genetic correspondence of *M. tuberculosis* 2-ponA genotypes with other common *M. tuberculosis* whole-genome genotyping methods such as Single Nucleotide Polymorphism (SNP, X-axis) and phylogenetic lineage (Y-axis) in 75 *M. tuberculosis* clinical isolates from the Public Health Research Institute (PHRI, Newark, NJ) strain bank. The identifier for each *M. tuberculosis* isolate is indicated by its IS6110 Restriction Fragment Length Polymorphism (RFLP) fingerprint (PHRI library). Each 2-ponA genotype family is indicated by a different color: genotype 0^T^ (n=22, black), 1^T^ (n=4, orange), 1 (n=14, red), 2 (n=16, blue), 3 (n=16, green), 4 (n=3, purple). 2-ponA genotypes are grouped as “clouds” with line connectors indicating the presence of genetic outliers within each group. The 75 *M. tuberculosis* isolates shown here are also listed in Table S2 together with their spoligotype identifier.

**Figure D:** Results of the TOP TB assay in discarded sputum samples of patients with suspected pneumonia spiked with decreasing numbers of *Mycobacterium bovis* Bacille Calmette-Guérin (BCG). **Top panel:** Analytical sensitivity in sputum specimens comparing TOP and Xpert MTB/RIF (*rpoB*) primers. By using only the primers and not the complete assay, the results shown may not accurately represent the analytical sensitivity of Xpert MTB/RIF. **Bottom panel:** Detection and quantification of *M. tuberculosis* amplicons using a capture-probe colorimetric assay.


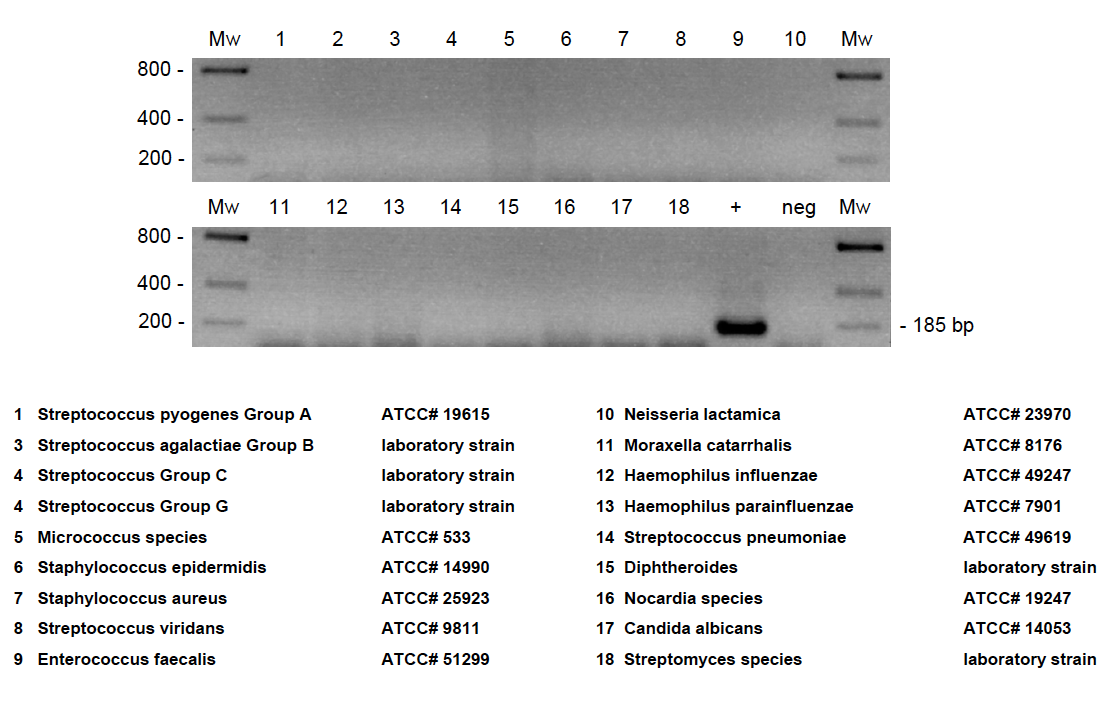


**Figure E**: Analytical specificity of 3-ponA primer tested with DNA from a set of 18 common respiratory pathogens and other microorganisms (10^8^ colony forming units/PCR).

**Figure F**: Specificity of TOP TB assay against non-tuberculous mycobacteria (NTM).

A) *In silico specificity*: The gene (*ponA1*) targeted by the TOP TB assay is large (~2,500 bp) and highly conserved providing 100% homology (~only 1 base difference every 500 base pairs) between all mycobacteria in the *M. tuberculosis* complex (*M. tuberculosis, M. africanum, M. bovis, M. microti, M. canetti, M. pinipedii, M. mungi, M. caprae, M. orygis*). As shown in the tables above, the *ponA1* of other mycobacteria and related bacteria are situated at a substantial genetic distance (15%) from *M. tuberculosis* (~1 base difference every 6 base pairs). By comparison, the 16rRNA gene of *M. tuberculosis* H37Rv and *M. kansasii* are 98.7% homologous (20 bp differences).

B) *In vivo* specificity: To date, 8/106 (7.5%) pulmonary TB suspects studied in Brazil (N=46) and Boston (N=60) had a positive culture for NTM. Of these, 6 were TOP-negative (3 *M. avium*, 2 *M. kansasii and 1 M. abscessus*) and 2 were TOP-positive (*M. abscessus* and *M. malmoense*). The clinical histories of both patients with discordant NTM results are suspicious for mixed mycobacterial infections (clinical data not shown).


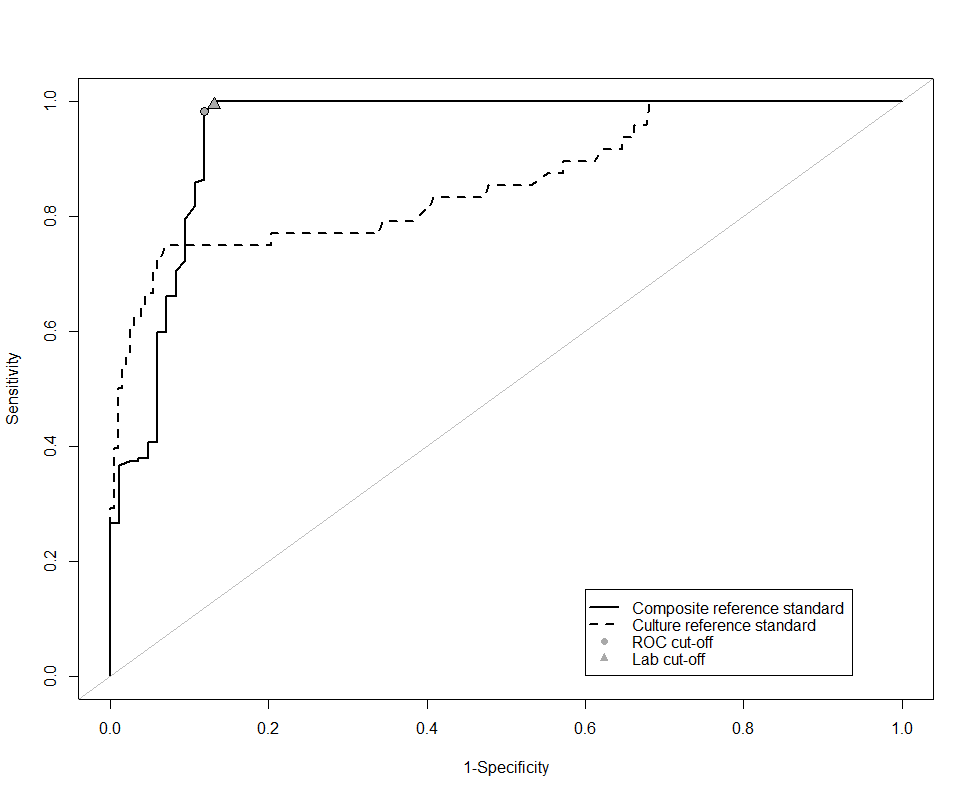


**Figure G:** ROC analysis of TOP OD values with either the composite reference standard (AUC=0.95) or culture (AUC=0.86) as the reference method.

ROC= Receiver operating curve; AUC= Area under curve

1. **Supplementary Tables**

**Table A**: Description of 3-ponA and 2-ponA primers. The two primer sets target two separate regions of *ponA1* (2,034 bp) that are 1,131 bp apart.

|  | Primer set 3-ponA (Diagnostic) | Primer set 2-ponA (Genotyping) |
| --- | --- | --- |
| PCR product length | 184 bp | 136 bp |
| Primer sequences | 5’-GACCGTTACCGAAGGGGCGTTGTTGG-3’  5’-ATCGGGCGGCACTGTCTCGGGAA-3’ | 5’-GGTCGGTGGTTATGCGGGTGTGCC-3’  5’-CAGGGTAATGGTGGTCGGGGGA-3’ |
| Internal stability (www.idtdna.com) | No primer-dimers  Delta G: -3.61 kcal/mole; Base Pairs: 2 | No primer-dimers  Delta G: -3.61 kcal/mole; Base Pairs: 2 |
| Primer location in *ponA1* (*M. tuberculosis* H37Rv Y980_0050) | 537 −> 720 | 1851 −> 1983 |
| Targeted DNA diagnostic feature | Differentiation of *M. tuberculosis* from non-tuberculous mycobacteria and other related microorganisms | Differentiation between *M. tuberculosis* into five major *ponA1* genotypes and sub-variants |

**Table B**: Results of genotyping a global collection of 75 *M. tuberculosis* clinical isolates banked at the Public Health Research Institute (PHRI, Newark, NJ) ordered according to 2-ponA genotyping results. The 75 isolates and their isolated DNA were selected from the PHRI TB Center strain collection, which includes more than 33,000 genetically characterized strains from global sources. The selection was based on geographic diversity and the strain’s genotype distinguished by Single Nucleotide Polymorphism (SNP) analysis, which catalogues the strains into three major principal genetic groups (1-3) and nine genetic clusters (I - VIII).

| # | 2-ponA genotype | Phylogenetic Lineage | SNP | RFLP | Spoligotype | PHRI lab number |
| --- | --- | --- | --- | --- | --- | --- |
| 1 | 0^T^ | 1 | I | IK | S00162 | 6464 |
| 2 | 0^T^ | 1 | I | BE | S00091 | 11677 |
| 3 | 0^T^ | 1 | I | HR36 | none | 12387 |
| 4 | 0^T^ | 1 | I | BE1 | S00015 | 13448 |
| 5 | 0^T^ | 1 | I | BE | S00681 | 13652 |
| 6 | 0^T^ | 1 | I | BE | S00121 | 13860 |
| 7 | 0^T^ | 1 | I | IP12 | S00197 | 14259 |
| 8 | 0^T^ | 1 | I | 1 | S00778 | 14328 |
| 9 | 0^T^ | 1 | I | 1 | S00806 | 14396 |
| 10 | 0^T^ | 1 | I | 1 | S00808 | 14426 |
| 11 | 0^T^ | 1 | I | *M. africanum* | none | 15140 |
| 12 | 0^T^ | 1 | I | MC17 | S01245 | 23060 |
| 13 | 0^T^ | 1 | II | W1 | S00030 | 27057 |
| 14 | 0^T^ | 1 | II | W | S00034 | 30463 |
| 15 | 0^T^ | 1 | II | W | none | 565 |
| 16 | 0^T^ | 1 | II | AM | S00034 | 4948 |
| 17 | 0^T^ | 1 | II | KY | S00199 | 10545 |
| 18 | 0^T^ | 1 | II | HE | S00199 | 13454 |
| 19 | 0^T^ | 1 | II | W615 | none | 15536 |
| 20 | 0^T^ | 1 | II | AR3 | none | 16689 |
| 21 | 0^T^ | 1/2 | IIA | HI | S00224 | 5116 |
| 22 | 0^T^ | 1/2 | IIA | 1 | S00199 | 10544 |
| 23 | 1^T^ | 1/2 | IIA | 1 | none | 13129 |
| 24 | 1^T^ | 1/2 | IIA | MY5 | none | 13141 |
| 25 | 1^T^ | 1/2 | IIA | NU | S00210 | 13984 |
| 26 | 1^T^ | 1/2 | IIA | GD139 | S00210 | 18270 |
| 27 | 1 | 1 | I | BE3 | S00035 | 11657 |
| 28 | 1 | 2 | IV | BE | S00650 | 13443 |
| 29 | 1 | 2 | IV | H | S00009 | 13571 |
| 30 | 1 | 2 | IV | H | S00028 | 13628 |
| 31 | 1 | 2 | IV | BE | S00075 | 14248 |
| 32 | 1 | 2 | IV | H | S00003 | 16015 |
| 33 | 1 | 2 | IV | KI | S00030 | 20907 |
| 34 | 1 | 2 | IV | C | S00009 | 21435 |
| 35 | 1 | 2 | IV | BE | S00650 | 22085 |
| 36 | 1 | 2 | IV | H55 | S00075 | 24991 |
| 37 | 1 | 2 | IV | C | S00030 | 31106 |
| 38 | 1 | 2 | IV | BE | S00009 | 13294 |
| 39 | 1 | 2 | V | I | S0072 | 30674 |
| 40 | 1 | 2 | V | V | S00003 | 31043 |
| 41 | 2 | 1/2 | IIA | LL | S00393 | 12578 |
| 42 | 2 | 2 | III | DG | S00035 | 28339 |
| 43 | 2 | 2 | IV | BE | S00075 | 7556 |
| 44 | 2 | 2 | IV | C28 | S00075 | 9319 |
| 45 | 2 | 2 | IV | C37 | S00919 | 15350 |
| 46 | 2 | 2 | IV | GH | S00273 | 17853 |
| 47 | 2 | 2 | IV | 1 | S00030 | 18410 |
| 48 | 2 | 2 | IV | H | S00031 | 21386 |
| 49 | 2 | 2 | IV | L | S00009 | 21479 |
| 50 | 2 | 2 | IV | H | S02278 | 31112 |
| 51 | 2 | 2 | IV | H6 | S00075 | 10443 |
| 52 | 2 | 2 | V | L | S00006 | 10999 |
| 53 | 2 | 2 | V | AH13 | none | 15552 |
| 54 | 2 | 2 | VI | CC | S00157 | 30558 |
| 55 | 2 | 3 | VII | U | none | 24493 |
| 56 | 2 | 3 | VII | M51 | none | 15601 |
| 57 | 3 | 2 | III | AU | S00491 | 8623 |
| 58 | 3 | 2 | III | BW33 | S00625 | 11607 |
| 59 | 3 | 2 | III | DK | none | 29669 |
| 60 | 3 | 2 | III | E | S00005 | 30855 |
| 61 | 3 | 2 | IV | C | S00028 | 19216 |
| 62 | 3 | 2 | IV | H | S00140 | 21179 |
| 63 | 3 | 2 | V | NS | none | 29915 |
| 64 | 3 | 2 | VI | J | S00080 | 31368 |
| 65 | 3 | 2 | VI | AI36 | S00192 | 12556 |
| 66 | 3 | 2 | VI | HP81 | none | 26838 |
| 67 | 3 | 3 | VII | GY | none | 31382 |
| 68 | 3 | 3 | VII | BF | none | 13108 |
| 69 | 3 | 3 | VIII | AF | S00002 | 9139 |
| 70 | 3 | 3 | VIII | AF89 | none | 16720 |
| 71 | 3 | 3 | VIII | H37Ra | s00001 | ATCC 25177 |
| 72 | 3 | 3 | VIII | H37Rv-9 | s00001 | ATCC 25618 |
| 73 | 4 | 1/2 | IIA | KQ | S00770 | 14255 |
| 74 | 4 | 2 | VI | P | S00086 | 13151 |
| 75 | 4 | 3 | VIII | H37Rv-7 | s00001 | ATCC 35837 |

RFLP= Restriction fragment length polymorphism

SNP= Single nucleotide polymorphism

**Table C**: Summary of clinical study.

|  | Mbarara, Uganda  N=261 |
| --- | --- |
| Study design | Prospective, cross-sectional clinical study to evaluate a new acid-fast bacilli smear method and Xpert MTB/RIF |
| Patients | Adult, hospitalized and ambulatory HIV-infected TB suspects |
| Specimen type and handling | Discarded sputum pellet processed for Xpert MTB/RIF. Pellet was washed prior to DNA extraction to remove NaLC/NaOH. Specimens were tested in a single batch in Uganda |
| Reference methods | Liquid culture (manual MGIT 960) and Xpert MTB/RIF |
| Other clinical and microbiological data available | Clinical information and results from sister sputum samples tested as part of parent clinical study |

NaCl= N-acetyl-L-cysteine; NaOH= sodium hydroxide

**Table D:** Breakdown of results of the Composite Reference Standard (CRS) in 261 pulmonary tuberculosis suspects in Mbarara, Uganda.

| **Composite Reference Standard Criteria** | | | | TOP TB assay | CRS interpretation | N |
| --- | --- | --- | --- | --- | --- | --- |
| Test 1 | Test 2 | Test 3 | Test 4 |
| Culture | Sequencing^1^ | Xpert MTB/RIF | AFB smear |
| + | + | + | +/− | + | MTB detected | 41 |
| + | + | − | +/− | + | MTB detected | 3 |
| + | − | + | +/− | + | MTB detected | 4 |
| − | + | + | +/− | + | MTB detected | 3 |
| − | + | − | − | + | MTB detected | 125 |
| − | + | − | − | − | MTB detected | 1 |
|  |  |  |  |  | Sub-total | 177 |
| − | − | − | − | + | MTB not detected | 11 |
| − | − | − | − | − | MTB not detected | 73 |
|  |  |  |  |  | Total | 261 |

AFB= Acid fast bacilli; MTB= *M. tuberculosis*

^1^ Refers to a positive 2-ponA genotype

**Table E**: Results of sensitivity analysis, using 100 randomly selected samples to obtain optimal cut-off for the TOP TB assay (OD=0.0795). Then remaining 161 samples are used to calculate sensitivity, specificity, positive predictive value (PPV) and negative predictive value (NPV).

| Diagnostic test | MTB detected  (N) | MTB not detected  (N) | Sensitivity | | Specificity | | PPV | | NPV | |
| --- | --- | --- | --- | --- | --- | --- | --- | --- | --- | --- |
|  |  |  | N | % (95% CI) | N | % (95% CI) | N | % (95% CI) | N | % (95% CI) |
|  | **33** | **128^1^** | **Culture reference standard** | | | | | | | |
| Xpert MTB/RIF ^2^  TOP TB assay | 34  114 | 125  47 | 30/32  31 | 94% (78, 99)  94% (80, 99) | 123/127  48 | 97% (92, 99)  35% (27, 44) | 30  31 | 88% (72, 96)  27% (19, 36) | 123  45 | 98% (94, 100)  96% (85, 99) |
|  | **111** | **50** | **Composite reference standard** | | | | | | | |
| Culture  Xpert MTB/RIF  TOP TB assay | 33  34  114 | 128  125  47 | 33  32/110  107 | 30% (22, 39)  29% (21, 39)  96% (91, 99) | 50  47/49  43 | 100% (93, 100)  96% (85, 99)  86% (73, 94) | 33  32  107 | 100% (89, 100)  94% (87, 97)  94% (87, 97) | 50  47  43 | 39% (31, 48)  38% (29, 47)  91% (80, 98) |

Definition of abbreviations: MTB= *Mycobacterium tuberculosis;* CI= Confidence interval; PPV= Positive predictive value; NPV= Negative predictive value

^1^ Includes patients with contaminated culture results

^2^ Two Xpert MTB/RIF results were missing and 4 had indeterminate result (N=259)

^3^ Composite Reference Standard (CRS) included *M. tuberculosis* culture, *M. tuberculosis* sequencing (e.g. 2-ponA genotyping), a NAAT other than TOP (e.g. Xpert MTB/RIF) and AFB smear.

1. **References for Appendix.**

1. Kacena KA, Quinn SB, Howell MR, Madico GE, Quinn TC, Gaydos CA. Pooling urine samples for ligase chain reaction screening for genital Chlamydia trachomatis infection in asymptomatic women. J Clin Microbiol. 1998;36(2):481-5. Epub 1998/02/18. PubMed PMID: 9466763; PubMed Central PMCID: PMC104564.

2. Madico G, Quinn TC, Boman J, Gaydos CA. Touchdown enzyme time release-PCR for detection and identification of Chlamydia trachomatis, C. pneumoniae, and C. psittaci using the 16S and 16S-23S spacer rRNA genes. J Clin Microbiol. 2000;38(3):1085-93. Epub 2000/03/04. PubMed PMID: 10699002; PubMed Central PMCID: PMC86346.

3. Madico G, Quinn TC, Rompalo A, McKee KT, Jr., Gaydos CA. Diagnosis of Trichomonas vaginalis infection by PCR using vaginal swab samples. J Clin Microbiol. 1998;36(11):3205-10. Epub 1998/10/17. PubMed PMID: 9774566; PubMed Central PMCID: PMC105302.

4. Pham DG, Madico GE, Quinn TC, Enzler MJ, Smith TF, Gaydos CA. Use of lambda phage DNA as a hybrid internal control in a PCR-enzyme immunoassay to detect Chlamydia pneumoniae. J Clin Microbiol. 1998;36(7):1919-22. Epub 1998/07/03. PubMed PMID: 9650936; PubMed Central PMCID: PMC104952.

5. Roymans RT, Onland G, Postma BH. One-day detection of PCR amplified Chlamydia trachomatis DNA in clinical samples: ELISA versus Southern blot hybridisation. J Clin Pathol. 1996;49(7):581-3. Epub 1996/07/01. PubMed PMID: 8813959; PubMed Central PMCID: PMC500575.

6. Denis M, Soumet C, Legeay O, Arnauld C, Bounaix S, Thiery R, et al. Development of a semiquantitative PCR assay using internal standard and colorimetric detection on microwell plate for pseudorabies virus. Mol Cell Probes. 1997;11(6):439-48. Epub 1998/03/17. doi: 10.1006/mcpr.1997.0139. PubMed PMID: 9500814.

7. Clark TG, Mallard K, Coll F, Preston M, Assefa S, Harris D, et al. Elucidating emergence and transmission of multidrug-resistant tuberculosis in treatment experienced patients by whole genome sequencing. PLoS One. 2013;8(12):e83012. Epub 2013/12/19. doi: 10.1371/journal.pone.0083012. PubMed PMID: 24349420; PubMed Central PMCID: PMC3859632.

8. Jones-Lopez EC, Namugga O, Mumbowa F, Ssebidandi M, Mbabazi O, Moine S, et al. Cough aerosols of Mycobacterium tuberculosis predict new infection: a household contact study. Am J Respir Crit Care Med. 2013;187(9):1007-15. Epub 2013/01/12. doi: 10.1164/rccm.201208-1422OC. PubMed PMID: 23306539; PubMed Central PMCID: PMC3707366.

9. Gutacker MM, Mathema B, Soini H, Shashkina E, Kreiswirth BN, Graviss EA, et al. Single-nucleotide polymorphism-based population genetic analysis of Mycobacterium tuberculosis strains from 4 geographic sites. J Infect Dis. 2006;193(1):121-8. doi: 10.1086/498574. PubMed PMID: 16323140.

10. Sreevatsan S, Pan X, Stockbauer KE, Connell ND, Kreiswirth BN, Whittam TS, et al. Restricted structural gene polymorphism in the Mycobacterium tuberculosis complex indicates evolutionarily recent global dissemination. Proc Natl Acad Sci U S A. 1997;94(18):9869-74. PubMed PMID: 9275218; PubMed Central PMCID: PMC23284.

11. Galagan JE. Genomic insights into tuberculosis. Nat Rev Genet. 2014;15(5):307-20. Epub 2014/03/26. doi: 10.1038/nrg3664. PubMed PMID: 24662221.

12. Naaktgeboren CA, Bertens LC, van Smeden M, de Groot JA, Moons KG, Reitsma JB. Value of composite reference standards in diagnostic research. BMJ. 2013;347:f5605. doi: 10.1136/bmj.f5605. PubMed PMID: 24162938.

13. Denkinger CM, Schumacher SG, Boehme CC, Dendukuri N, Pai M, Steingart KR. Xpert MTB/RIF assay for the diagnosis of extrapulmonary tuberculosis: a systematic review and meta-analysis. Eur Respir J. 2014;44(2):435-46. Epub 2014/04/04. doi: 10.1183/09031936.00007814. PubMed PMID: 24696113.
